# Mental capacity—why look for a paradigm shift?

**DOI:** 10.1093/medlaw/fwac052

**Published:** 2023-01-13

**Authors:** Alex Ruck Keene, Nuala B Kane, Scott Y H Kim, Gareth S Owen

**Affiliations:** Barrister, Visiting Professor, King’s College London, Dickson Poon School of Law, UK; Mental Health Research UK MD(Res) scholar, King’s College London, Department of Psychological Medicine, Institute of Psychiatry, Psychology and Neuroscience, London, UK; Senior Investigator in the Department of Bioethics, National Institutes of Health, USA; Reader/Honorary Consultant Psychiatrist. King’s College London, Department of Psychological Medicine, Institute of Psychiatry, Psychology and Neuroscience, London SE5 8AF, UK

**Keywords:** Capacity assessment, Convention on the Rights of Persons with Disabilities, Decision-making capacity

## Abstract

Challenges to the legitimacy of mental capacity over the past 10 years have been spearheaded by the Committee on the Rights of Persons with Disabilities, the treaty body for the UN Convention on the Rights of Persons with Disabilities (CRPD). This challenge has been asserted to have produced a ‘paradigm shift’. In this article, we examine why that interpretation has had such limited traction in the legal policy arena, and whether it should have traction. We also analyse whether the Committee has subtly but importantly shifted its position. We then develop an argument that the true goal, compatible with the CRPD, is the satisfactory determination of whether a person has or lacks mental capacity to make or take a relevant decision. Our article contextualises multi-disciplinary, research-informed guidelines designed as a contribution to satisfactory determination. While our article is based upon the position in England and Wales, we suggest that our conclusions are of wider application.

## I. INTRODUCTION

This article represents the legal fruits of the contested capacity workstream of a five-year Wellcome Trust-funded project, Mental Health & Justice. The multidisciplinary^1^ workstream started with two core propositions:

1. That there are circumstances under which individuals are (either temporarily or permanently) unable to make a decision, and that legal mechanisms will always be required to respond to such situations;
2. There are many cases which arise under current legislative regimes designed for this purpose whose resolution is difficult, and where outcomes at present—whether inside or outside the court room—all too often seem unsatisfactory.

We have been reinforced in our conviction as to both propositions, both by the work that we have done during the project and by external developments during its life. This article serves to address the first proposition; it accompanies multi-disciplinary, research-informed guidelines^2^ designed as a modest contribution towards resolving the problem captured by the second.

## II. THE LEGITIMACY OF MENTAL CAPACITY

Had this article been written 15, or even 10, years ago, addressing the legitimacy of the concept of mental capacity would have seemed both odd and unnecessary. It would perhaps have been necessary to do some explanatory work to set out the functional model of mental capacity underpinning the Mental Capacity Act 2005 (‘MCA 2005’), the governing legislation in England and Wales, as well as many other late 20th legislative frameworks. In particular, it would have been necessary to do some explanatory work to make clear how this concept—ie focusing upon the ability of the person in question to make the decision in question at the time in question—was different from status-based or outcome-based models. But there could have been no sensible challenge to the idea that, whether permanently or temporarily, a person might not be able to make a decision.

Over the past 10 years, however, there has been a sustained challenge to the concept of mental capacity. That challenge is not just to the way in which it is assessed, or legal responses where the individual in question lacks capacity (applying whatever mechanism is used) but to the very legitimacy of the concept. That challenge has been spearheaded by the Committee on the Rights of Persons with Disabilities, the treaty body for the UN Convention on the Rights of Persons with Disabilities (the ‘Committee’ and the ‘CRPD’, respectively). As we discuss further below, the Committee’s challenge overlaps with other concerns that have been raised in relation to the concept. However, given that it relates to a UN Convention signed by the majority of States—including the UK—the Committee has taken the challenge out of halls of academia and squarely into the legal policy space. For better or worse, the Committee has also chosen the terrain upon which the challenge is to be determined by the way in which it has approached matters.

Article 12 of the CRPD enshrines the right to legal capacity, Article 12(2) providing that States Parties ‘shall recognise that persons with disabilities enjoy legal capacity on an equal basis with others in all aspects of life’, and 12(3) providing that such States Parties ‘shall take appropriate measures to provide access to persons with disabilities to the support they may require in exercising their legal capacity’. The Committee deliberately chose Article 12 to be the subject of its first General Comment, as ‘[o]n the basis of the initial reports of various States parties that it has reviewed so far, the Committee observes that there is a general misunderstanding of the exact scope of the obligations of States parties under article 12 of the Convention’.^3^ The Committee considered^4^ that there had been ‘a general failure to understand that the human rights-based model of disability implies a shift from the substitute decision-making paradigm to one that is based on supported decision-making’,^5^ and it set out expressly to frame its own interpretation of Article 12.^6^ Much of the General Comment is then dedicated to the concept of ‘supported decision-making’, and much ink has been spilled on the subject.^7^ However, for present purposes, of more relevance is the distinction that the Committee drew in General Comment 1 between legal capacity and mental capacity. As the Committee noted at paragraph 13:

*Legal capacity and mental capacity are distinct concepts. Legal capacity is the ability to hold rights and duties (legal standing) and to exercise those rights and duties (legal agency). It is the key to accessing meaningful participation in society. Mental capacity refers to the decision-making skills of a person, which naturally vary from one person to another and may be different for a given person depending on many factors, including environmental and social factors.*

Although this passage is not entirely free from ambiguity,^8^ that there is, or should be, a distinction between a person’s functional abilities and the legal status afforded that person’s exercise of those abilities is not controversial. Indeed, the distinction between the two concepts has now expressly been recognised in English law, which is perhaps of some note given the very limited status afforded by the CRPD and the Committee before the English courts.^9^ In *An NHS Trust v X*,^10^ Sir James Munby identified the distinction between legal capacity—a question of ‘personal status, irrespective of any impairment or disturbance of mental functioning’—and mental capacity, which has ‘nothing to do with personal status; it is all to do with impairment or disturbance of mental functioning’. A person in England and Wales will always be recognised as a rights holder, irrespective of their mental functioning.^11^ However, and in common with many, if not most, jurisdictions, the functional model underpinning the MCA 2005 and equivalent legislation proceeds on the basis that there will be circumstances (for anyone, at any point) at which it simply is not possible to support the person’s mental functioning so as to enable a decision with legal consequences to be recognised.

Where things become more difficult is where the Committee went next in General Comment 1 in attacking the linkage between mental capacity and legal capacity outlined (in English terms^12^) immediately above. The Committee asserted that mental capacity was not ‘as commonly presented, an objective, scientific and naturally occurring phenomenon. Mental capacity is contingent on social and political contexts, as are the disciplines, professions and practices which play a dominant role in assessing mental capacity’.^13^ Not only did the Committee attack the older models of capacity such as that based on status, ie that a diagnosis of an impairment automatically meant that the individual’s decisions could not be regarded as legally valid, the Committee also attacked the functional model. The Committee considered the functional approach to be flawed in part because ‘it presumes to be able to accurately assess the inner-workings of the human mind and, when the person does not pass the assessment, it then denies him or her a core human right—the right to equal recognition before the law’.^14^

The Committee’s interpretation of Article 12 has been influential in academic circles.^15^ We suggest that this reflects both the involvement of academics in framing General Comment 1^16^ and also the fact that the General Comment 1 overlaps with other, longer-standing, academic challenges to the validity of the concept of capacity.^17^

However, the Committee’s interpretation has had a very different reception in the legal policy space. It has, for instance, not swayed the courts in England & Wales. In the first case concerning mental capacity to reach the Supreme Court, *A Local Authority v JB*,^18^^,^^19^ the argument was advanced in the context of sexual activity that the requirement for ability to consent to such activity (and the giving of consent throughout) created ‘a separate standard or test of capacity for people with disabilities’, contrary to the obligations imposed by Article 12(2) CRPD. Lord Stephens, giving the unanimous judgment of the court, considered that the argument failed at the first hurdle:

*There is no separate standard or test for persons with disabilities. The fact that the other person must have the ability to consent to the sexual activity and must in fact consent before and throughout the sexual activity applies to everyone in society.^20^*

Nor has the Committee’s interpretation of Article 12 swayed the German Federal Constitutional Court,^21^ the European Court of Human Rights,^22^ or courts further afield, all of whom have adhered to a functional model of mental capacity. Nor, it should be noted, has it swayed all other parts of the UN Human Rights system.^23^

The resistance by these bodies—and, indeed, legislative bodies^24^—could simply be portrayed as wrong-headed. But we suggest that there is more at work here. In particular, and in the section that follows, we suggest that there are four critical reasons why the interpretation in General Comment 1 has had such limited traction, all of which speak also to whether it **should** have traction.

## III. CHALLENGING THE CRITIQUE OF MENTAL CAPACITY

To the best of our knowledge, our article is the first to draw together the four challenges to the Committee’s critique that we develop below,^25^ and also the first to do on the basis of sustained, multi-disciplinary engagement with both the ‘micro’ level of capacity determinations in practice and the ‘macro’ level of the framework within those determinations sit.

The first challenge to the Committee's critique is that legal systems predicated upon the late twentieth century model of functional, decision-specific capacity do not, in fact, proceed on the basis that capacity is some form of objective, measurable, phenomenon. In other words, they do not pretend to be able accurately to assess the inner workings of the human mind—if by ‘accurately’, the assertion is that it is possible to identify some form of mind-reading measuring device.^26^

In this regard, and although this is speculation, the CRPD Committee may potentially have had in mind tests or rating scales (such as IQ or the Mini-Mental State Exam), as these are the most commonly used—and misunderstood—tools in play in the capacity field. It is possible to produce a correlation that a low MMSE score corresponds reliably with impaired decision-making capacity. However, correlation estimates are based on groups of individuals.^27^ There is no test score which, in itself, justifies the presence or absence of a ‘specific’ person’s ability to make a ‘specific’ decision. Therefore, models such as the MCA 2005 proceed on the basis that there must be a reasoned justification given for the conclusion reached as to why a legal test is met. The MCA 2005 and similar legislative frameworks are predicated upon mechanisms to require structured assessment by those asserting that the person in question does not have the mental capacity to make the relevant decision. In other words, their focus is upon process, rather than outcome, a point to which we return below.

The second challenge to the critique is that those framing^28^ and applying legislation such as the MCA 2005 were—and are—aware that capacity is ultimately in part a social construct. Nowhere is this clearer than in *A Local Authority v JB*,^29^ a case which, as we have seen above, concerned capacity and sexual relations. Before the Supreme Court, the question was whether the man in question, JB, had to be able to understand, use and weigh the information that any prospective sexual partner must be able to, give, and maintain consent to any sexual activity he was initiating. The expert evidence before the court relating to JB was to the effect that he could not understand, use and weigh this information about the partner. But—critically—the evidence that he could not process this information was legally insufficient to ground a conclusion that he lacked the capacity to do so for purposes of the MCA 2005.^30^ That required consideration of whether the information about the other’s consent was ‘relevant information’ for purposes of the test contained within sections 2 and 3 MCA 2005. It further required consideration of what consequences were to be seen as reasonably foreseeable, as such consequences fall within the definition of ‘relevant information’.^31^ That question was a legal question, to be answered by reference not solely to clinical considerations, but by application of legal principles. These principles included importantly, obligations imposed upon the Supreme Court as a public authority to act compatibly with the European Convention on Human Rights, which led Lord Stephens (giving the sole judgment) to identify at paragraph 92 that ‘the court as a public authority, in determining what information is relevant to the decision, **must** include reasonably foreseeable adverse consequences for P and for members of the public’. The word ‘must’, emphasised here, reflects a normative framing, reflecting societal values, as opposed to the uncovering of some objective ‘truth’.

Further, Lord Stephens rejected the argument advanced by the Official Solicitor on behalf of JB that to include the information ‘*imposes a discriminatory cerebral analysis on the potentially incapacitous*’, holding at paragraph 96 that:

[…] *As the Court of Appeal observed, at para 96, “amongst the matters which every person engaging in sexual relations must think about is whether the other person is consenting” (emphasis added). If that is properly viewed as cerebral or as involving a degree of analysis, a decision to engage in sexual relations is necessarily cerebral or analytical to that extent.*^32^

The change in the characterisation of the information relevant to the decision between Roberts J (who had excluded the requirement for the other’s consent) and the Court of Appeal (which included it) meant that, by the time that the Supreme Court came to consider the position, at least one person had been found to lack capacity who might previously be found to have had it.^33^ Some might argue that this demonstrates precisely the problems with the legitimacy of the concept of mental capacity that the CRPD Committee has addressed in General Comment 1. The counter-argument is that transparency as to and scrutiny of the nature of the decision in question of the kind undertaken by the Court of Appeal and the Supreme Court is one way in which society can test whether decisions are being framed in a way which balances all the interests at stake.^34^ Such a process means that the inherently normative considerations lurking behind considerations of capacity can be brought fully out into the open.^35^

In other words, the approach of the Supreme Court in *JB* can be seen as an illustration of the fact that, while mental capacity may be a social construct, it is a construct that serves vital and legitimate functions. Those functions include (amongst others): (i) the delineation of the existence, scope and limit of the State’s duties to secure the vital interests of its citizens, (ii) identification of when the State is either justified in intervening in or even required to intervene in relationships between those citizens, and (iii) identification of when individuals should be held to the consequences of their actions—whether for themselves or others, including both in the civil sphere (the formation of a contract, for instance) and in the criminal sphere.^36^ It is a construct which may well be open to the criticism that it is philosophically inadequate, but in that, it is not unusual^37^; indeed, the very fact that it does not purport to philosophical perfection—and hence is an incompletely theorised agreement—may, in and of itself, carry its own virtues. Indeed, a particular virtue of the construct might be thought that it enables agreement to be reached in a liberal society about how to act amongst those who may have very different motives for acting.^38^

By way of example, we return to the concept of capacity in the context of sexual relations. This is a particularly fruitful area to examine, we suggest because it engages all of the potential functions of mental capacity addressed in the paragraph above. We note that it is telling—and important—that both those who might stereotypically be said to be motivated by a desire to protect and those stereotypically be said to be motivated by a desire to empower seem, ultimately, to reach for the same construct of capacity. As one of the leading advocates for the General Comment 1 position has noted in the course of developing a model for sexual relations compliant with Article 12:

[i]*t is, of course, important to ensure that all parties in any sexual activity are agreeing to that sexual activity. That agreement cannot exist without the parties having the decision-making skills necessary to* ***understand****, to some extent, what the nature of the sexual activity is* (emphasis added).^39^

It is difficult, in truth, to see how this statement does not imply support for a functional test, as well as recognition of an underlying—valid—social function being served by some notion of mental capacity.

The third challenge to the critique, building on the second, relates directly to the legitimacy of the challenge being mounted by the Committee. The term ‘legitimacy’ is one which encompasses a number of aspects, all of which relate to the wide and fundamental range of social functions performed by the concept of mental capacity.

We start with the very formulation of the challenge by the Committee. It is important to unpick whether (i) the Committee is challenging the social functions performed by the concept of mental capacity, or (ii) whether the Committee accepts their significance, but is proposing an alternative mechanism by which those functions be addressed. That the Committee has, to date, simply focused on the challenge to the concept makes answering this question difficult. The Committee might say that it is not its function to identify how States are to discharge their obligations under Article 12 CRPD and abolish reliance upon the functional concept of capacity.^40^ However, on one, unsympathetic, view, it might be said that in so doing, the Committee has sought to define away the problem of mental capacity rather than addressing the consequences of its removal. Is that approach legitimate?

Moving ‘up’ a level in terms of legitimacy, and given that the Committee is asking the relevant bodies within States Parties (executive, legislative, and judicial) to adopt an approach which denies the possibility of mental incapacity—in any and all circumstances, and for all purposes—we suggest that it is appropriate to ask upon what basis that request is made.^41^

It is striking in this regard to note, for instance, that a former member of the UN Human Rights Committee, Gerald L Neuman, has argued that ‘*the Committee’s absolutism endangers many of the people living with moderate or severe dementia whom it supposedly benefits’*.^42^ And it is, we suggest, of no little importance that the CRPD Committee and the UN Human Rights Committee—institutionally independent of each other, but both part of the UN human rights system—do not appear to see eye to eye as regards the legitimacy of mental capacity.^43^ While neither body holds a formal democratic mandate,^44^ and the ‘flat’ structure of the Committees means that there is no hierarchy between the two, it might be suggested that the Human Rights Committee is better placed to speak to the ‘whole systems’ change of the nature proposed by the CRPD Committee.

Equally, if not more importantly, we suggest that it is appropriate to ask whether acceding to the ‘hard-line’ approach advocated in General Comment 1 would benefit all of those who upon whose behalf the Committee seeks to speak, and whether those driving hardest in this direction are sufficiently representative of what might be called their constituency—ie how much they are truly reflecting the ethos of the CRPD of ‘nothing about us without us’. This takes us into difficult waters (which we have experienced directly) and circumstances where service users who set out positions which are insufficiently aligned to the hard-line position are, in effect, told that they are wrong.^45^ We note also in this regard the work of Anne Plumb, who identifies as a ‘mental health system survivor’, and who has written powerfully about how ‘the premise behind the CRPD that all of us are able *at all times*, given support, to express our wishes is flawed’, and challenged the ‘tak[ing] of service-users/survivors *out of the frying pan of psychiatry and state provision and into the fires of libertarian ideology*’ (emphases in original).^46^

Further, and, while Gerald L Neuman does not (as far as we are aware) describe himself as a person within the scope of the CRPD, we suggest that his observation in relation to the ‘absolutism’ of the Committee is not one that can be easily dismissed. Put another way, is it truly self-evident that an approach which may (or may not) meet the concerns of those most troubled by psychiatrists seeking to act in the context of mental distress applies equally in relation to all conditions which might affect decision-making ability?^47^

Probing the implications of the Committee’s approach is important given that any model which is to replace the functional model of capacity must apply across the full spectrum of the law, and to have the same consequences across every aspect of the law.

If the consequences in one context are unacceptable, that is a red flag for caution. In this regard, it should be noted that the Committee’s approach can lead to consequences in the criminal field that appear—at a minimum—challenging even by reference to the CRPD itself, let alone any external considerations. As the Australian Law Reform Commission noted in response to ‘concerns that functional tests of ability may present inappropriate barriers to the exercise of legal agency’, ‘the integrity of a criminal trial (and, arguably, the criminal law itself) would be prejudiced if the defendant does not have the ability to understand and participate in a meaningful way. It may also breach the person’s human rights by denying them a fair trial, implicating arts 12 and 13 of the CRPD’.^48^

The fourth and final challenge to the critique is that it is not obvious how removing the concept of capacity would do anything other than move the terrain for disagreement. Some might consider that the right approach is to proceed on the basis of the person’s apparent will and preferences.^49^ However, on such an approach, there would equally be cases with room for reasonable disagreement about what outcome would be dictated by proceeding on the basis of will and preferences.^50^ What defence would there be then at that point against the charge of arbitrariness in outcome^51^?

## IV. A CHANGE OF TACK FROM THE COMMITTEE?

In light of all the matters set out above, we suggest that the fact General Comment 1 has never been accompanied by the in-depth guidance promised at the time of its publication is telling. Indeed, it is important to identify that the Committee has, subsequently, articulated a position that suggests that the position adopted in that General Comment was untenable.

In its concluding observations under the second report of Australia upon compliance with the CRPD,^52^ and recalling General Comment 1, the Committee recommended (in paragraph 24) that Australia implemented a nationally consistent supported decision-making framework, as recommended in the Australian Law Reform Commission (‘ALRC’)’s 2014 report, ‘Equality, Capacity and Disability in Commonwealth Laws’.^53^ The ALRC’s report represents, by some margin, the most detailed law reform proposal advanced to date to seek to ‘operationalise’ the CRPD. For present purposes of particular importance is that is based upon the premise—at paragraph 3.48—that, contrary to the position set out in General Comment 1:

*with appropriate safeguards, and a rights emphasis, there is no ‘discriminatory denial of legal capacity’ necessarily inherent in a functional test* [of decision-making capacity, or ‘ability’ as the ALRC proposed] *—provided the emphasis is placed principally on the support necessary for decision-making and that any appointment* [of a decision-maker] *is for the purpose of protecting the person’s human rights.*^54^

It might on one view be thought slightly surprising that the Committee should endorse a model based on a premise so directly contrary to that set out in General Comment 1. It has not publicly repudiated the Comment, although its former chair has described the Committee as perhaps having been ‘naïve’ to take on Article 12 for its first General Comment.^55^ One explanation may be that the composition of the Committee changed post-2014, to include Professor Rosemary Kayess,^56^ who had been a member of the ALRC’s advisory committee for its Report.

However, whatever the precise ins- and outs- of the position, and in the context of a debate which has become characterised more by heat than by light, the endorsement by the Committee of the ALRC’s approach can be taken as a constructive, operational, step forward, in keeping with the general premises of the CRPD itself.

## V. THE CONCEPT OF CAPACITY WITHIN THE MENTAL CAPACITY ACT 2005: THE ‘DIAGNOSTIC’ THRESHOLD

To recap, we suggest that the concept of mental capacity—that of tying legal decisional authority to a functional model of decisional capacity—is a fundamentally legitimate one. In other words, as a concept is not discriminatory, but a legitimate response to situations which may arise, at any point, in relation to anyone, disabled or otherwise. Its application in practice may be a different question, which we address further below.

Before we get there, though, we need to address one further potential ‘existential’ challenge to the model of capacity embodied in the Mental Capacity Act 2005. The legal definition of mental capacity within England and Wales (at least for purposes of the MCA 2005^57^) contains within it—in section 2(1)—the requirement that the inability to make the relevant decision is caused by an impairment of, or disturbance in the functioning of, their mind or brain.^58^

In the Law Commission’s work in the 1990s, it had recognised that the arguments for and against requiring a ‘mental disability’ to be established before someone is deemed to lack capacity were finely balanced.^59^ Ultimately, the Law Commission had concluded that such a hurdle would serve a useful gate-keeping function, to ensure that decision-making rights were not taken over prematurely or unnecessarily. It further, ultimately concluded that the protection offered by a diagnostic threshold outweighed any risk of prejudice or stigma affecting those who need help with decision-making.^60^

There are undoubtedly arguments as to whether this requirement is discriminatory by reference to the CRPD,^61^ and we note that Ireland, which has sought to enact legislation compliant with the CRPD, has enacted (but not yet brought into force) mental capacity legislation which does not include an impairment or disturbance provision.^62^ One of the draft Codes of Practice accompanying this legislation makes it express, in consequence, that ‘[i] is not a requirement to identify a cause or reason for a change in the relevant person’s capacity’.^63^ One may doubt whether, in practice, the courts (in particular) will find it acceptable to proceed to a conclusion that a person lacks (legal) capacity to make a decision without any’ explanation.^64^ And, as noted by one member of the Service User Advisory Group for the present project,^65^ identification of whether the person has an impairment or disturbance in the functioning of their mind or brain allows consideration of what steps could be taken to resolve the problem.

From a different direction, we also note that the courts in England and Wales have recognised that the causes of incapacity may sometimes not easily be captured by the so-called ‘diagnostic’^66^ test. In the creation of a jurisdiction over those who have capacity applying the MCA 2005 but are in some way seen to be vulnerable,^67^ the High Court has expressly sought to fill a statutory gap left by the MCA 2005.

The time may well therefore have come to revisit precisely what role the so-called ‘diagnostic’ test plays within the law in England and Wales. Given that the MCA 2005 enshrines a statutory concept of mental capacity, such revisiting would ultimately be one for Parliament, and we suggest, rightly so given the implications that redrawing the lines might have. And, even if ultimately those lines were to be redrawn to be akin to those (say) in Ireland to contain a purely functional test, there will remain a need to assess and determine whether the person does or does not have the material capacity/ability and how those assessments are justified.

## VI. CAPACITY ASSESSMENT: WHAT IS THE GOAL?

As noted in Section II above, we suggest that the CRPD Committee set up a straw man in General Comment 1 in asserting that mental capacity is some form of natural phenomena which is possible to accurately measure, in the same way that it might be possible to measure a person’s blood pressure or blood type. It may, in future, be possible for neuroscientific developments to provide context for and possibly indicators as to the person’s ability to make decisions, but, at least in a jurisdiction such as England and Wales, ultimately: (i) those markers will need to be judged by reference to a non-medical standard: ie what the law considers to constitute decision-making ability and (ii) any such markers are very unlikely to be able to give definitive assistance as to the person’s ability to make a specific decision at a specific point in time.

That there is—and is unlikely ever to be—a ‘measure’ which determines definitively whether a person has or lacks mental capacity to make a decision means that the goal of capacity assessment must be to have a legitimate process by which such assessment can be undertaken, and standards by which the outcome of that assessment recorded. Within that process, we also consider it important that—in the way that the Supreme Court did in *JB—*there is an open recognition both that capacity assessment has a normative aspect and openness in the application of those norms by those charged with carrying out assessment.

Indeed, in reality, we suggest that such meets the goal of ‘objectivity’ sought by the CRPD Committee. In this context, our approach is predicated upon the position that ‘objectivity’ is not, and cannot be, code for ‘truth’ in the sense of correspondence to naturally occurring ‘things’ or ‘facts’ but rather stands as the opposite of arbitrariness and illegitimacy.^68^ The judges of the Court of Protection recognise this, speaking of the need for capacity assessment which is ‘detached and objective’.^69^

A linked challenge to capacity assessments, implicit in General Comment 1, but a familiar theme in academic literature, is that they simply reflect the view of the assessor. At one level, that is self-evidently true,^70^ but that does not, in and of itself, need to lead to the nihilist conclusion that assessment is impossible, or incapable of producing a result which can legitimately be relied upon to have legal effect.

To escape the echo chamber of capacity law debates for a moment, we can perhaps best illuminate this proposition by the different approach taken to two thorny areas where analogue reality may have to be reduced to a legally binary answer.

In the first, identifying how long a person with a terminal condition may live has proven so difficult—and giving rise to such obvious injustices—that an administrative mechanism dependent upon a prognosis of 6 months has been abandoned.^71^ In other words, it has proven possible effectively to circumvent the problem of giving a binary answer, and seen as necessary to do so to achieve a more just outcome.

By contrast, in a very different, but equally difficult area, it has not been proven possible to avoid the need to give a legally binary answer: assessing the age of young asylum-seekers. As identified by the Court of Appeal in *BF (Eritrea) v Secretary of State for the Home Department*^72^:

*The problem is that in the case of many young asylum-seekers there is no objective way of establishing their age. They can produce no documentary evidence of their date of birth;^73^ they may genuinely not know it; and even if they do they have an obvious incentive to misrepresent it. There are no medical or scientific means for establishing the age of a young person with any precision. In such cases the necessary determination of whether he is over or under 18 will have to be made on the basis of a subjective assessment. (I should say that in in this context "subjective" is not dyslogistic* [i.e. not a term being used negatively]*: it means only that the person making the assessment has to make a judgement without the benefit of documentary or other objective evidence.)*^74^

In the face of these difficulties,^75^ English law has developed an approach to determining age—so-called *Merton-*compliant assessments.^76^ As the Court of Appeal noted, in observations which might be thought to be equally relevant to consideration of mental capacity,^77^ such assessments ‘are sometimes described as “objective”, but it is more accurate (at least in the typical case where the interview does not provide any objectively verifiable information) to regard them as providing a sophisticated and disciplined form of subjective assessment’.

What, therefore, does a ‘sophisticated and disciplined form of subjective assessment’ look like for purposes of determining whether a person has, or lacks, capacity to make a specific decision or decisions? It could be seen simply as the exercise of clinical judgment,^78^ but we are mindful that the assessment of capacity is not limited to clinicians. We can do no better in this regard than quote the judgment of Bell J sitting in the Supreme Court of Victoria in *PBU v Mental Health Tribunal and Melbourne Health; NJE v Mental Health and Bendigo Health*^79^ who, at paragraph 201, identified that:

*The fundamental principles of self-determination, freedom from non-consensual medical treatment and personal inviolability, and the equally fundamental principles behind the right to health, are most respected by capacity assessments that are criteria-focussed, evidence-based, person-centred and non-judgmental. Such assessments engage with the demand (or plea) of the person to be understood for who they are, free of pre-judgment and stereotype, in the context of a decision about their own body and private life.*^80^

Not only does this paragraph crystallise the critical features of a process that seeks to takes an objective approach to a subject,^81^ but it also captures the wider obligations that are in play in such circumstances, obligations which are sometimes underplayed by those who seek to challenge the legitimacy of the concept of mental capacity. When it comes to the person’s own interests,^82^ those obligations are not to be found within the Mental Capacity 2005 (or indeed, any equivalent legislation in other jurisdictions) but external to that Act. In legal terms, those obligations are to be found enshrined in regional and international human rights conventions, requiring states to do more than simply refrain (for instance) from taking life, but rather to take active steps to secure life in the presence of real and immediate risk.^83^ Conventionally, at least in the context of physical health, the extent of those steps will be dictated in part by whether the person is said to have capacity to make the decision to accept or refuse the measures proposed.^84^ Any challenge to the legitimacy of the concept of mental capacity must—we suggest—propose an equally legitimate basis upon which to identify when the state is justified in accepting a refusal of life-saving treatment as determinative.

Drawing the threads together, therefore, we suggest that the goal of capacity assessment is to produce a satisfactory determination: in other words, a determination which in a transparent fashion explains why it is considered that the person has or lacks the capacity to make the decision in question, with a level of detail commensurate to the gravity of the intervention and the urgency of the situation. Amplifying this, and contextualising our own contribution to the process^85^:

- We could have used the term ‘defensible’ determination instead of ‘satisfactory’, but we have deliberately chosen to avoid the former term because it can be conflated too easily with ‘defensive’, a term which is both too negative and too limited a set of connotations. ‘Satisfactory’, by contrast, is intended to convey that it is possible to reach a determination which has both legal and ethical legitimacy, and is grounded in realities pertaining both to the person’s functional abilities at that point in time, and the decision options as they actually exist;
- Transparency is critical for two reasons. The first is that it enables individual accountability, by allowing either the person or anyone else more easily to challenge the outcome of the determination reached. The second is that—at a systems level—transparency is the best way to enable patterns of practice (good or bad) to be identified. A significant part of our research on the project has been in support of this goal,^86^ and we suggest that it has the potential to improve significantly the granularity of assessment and recording;
- We make deliberate reference to the potential for a person to ‘have’ as well as to ‘lack’ capacity to make a specific decision. A conclusion that a person has capacity to make a decision can have just as significant a set of consequences for them (and others) as a conclusion that they lack capacity;
- Tailoring the detail required to the gravity of the intervention and the urgency of the situation is at one level simple pragmatism, but also reflects the necessary calibration between procedural rigour and the nature of the interests at stake. Expressly highlighting urgency also allows questions to be asked as to whether the situation was, indeed, genuinely urgent.

In English law, at least, it is possible to get a legally definitive answer to the question of whether a person has or lacks capacity to make a specific decision or decisions because a Court of Protection judge can declare (in relation to a person aged 16 or above) precisely that: section 15(1)(a) Mental Capacity Act 2005. The fact that it is possible, as a matter of law, for a judge to place definitively a person in the category of ‘having’ or ‘lacking’ capacity to make a decision means that satisfactory assessment in the English context could be said to equate, narrowly, to an assessment which can satisfy a judge. Where cases come to court, it might be said that a different set of considerations come into play as regards what society can expect as regards the determination of capacity. In particular, we might want to ask such questions as to whether those who are appointed as judges can (in effect) be trusted to make such decisions, and whether they are armed with the right tools for the job.^87^ We interrogated the workings of the Court of Protection in the first substantive paper to emerge from our research. That study concluded that ‘whilst the Court of Protection is still on a learning curve, its work provides a powerful illustration of what taking capacity seriously looks like’.

However, the vast majority of situations where consideration is being given to capacity do not come anywhere near the courtroom. Rather—in most cases in England and Wales^88^—the question is whether the relevant person has (i) taken reasonable steps to establish whether the individual lacks capacity in relation to the matter and (ii) reasonably believes that they lack capacity. At that point, they will be protected from liability if they proceed to carry out an act of care or treatment which they reasonably believe to be in the individual’s best interests (section 5 of Mental Capacity Act 2005). Concepts such as ‘reasonable steps’ and ‘reasonable belief’, familiar in English law, place a weight both upon individual decision-makers, but also, for these purposes, more broadly upon the system as a whole to enshrine the systems and processes to ensure—insofar as sensibly possible—that they support those decision-makers to think in ‘reasonable’ ways. The research-informed guidelines that have been produced by the team, publicly accessible at https://capacityguide.org.uk serve as a practical contribution to achieving that goal.

## VII. CONCLUSION

The term ‘paradigm shift’ is very regularly used in relation to the CRPD.^89^ However, in science, ‘paradigm shifts’ do not occur until a new theory achieves legitimacy as an explanatory tool which displaces the previous account.^90^ Looked at through this light, we suggest that it is, in fact, the functional approach to mental capacity which has been a paradigm shift, entirely supplanting in legitimacy either ‘outcome-’ or ‘status-’ based approaches. Linked to this—and undoubtedly spurred on by the CRPD and the Committee^91^—has been a recognition that a conclusion that a person cannot be found functionally to lack capacity to make a specific decision unless appropriate support has been offered without success.

The functional model of capacity is undoubtedly imperfect, but there has, however, yet to be an approach put forward by the Committee which has equivalent—or greater—legitimacy to it. In Kuhnian terms, we suggest that it has not therefore been displaced as the paradigm—a point which we suggest the Committee may now implicitly have recognised.

The Committee’s challenge has been hugely important in jolting jurisdictions such as England and Wales out of complacency and driving thinking towards how to support decision-making, and how to be more attentive to the place of wishes and feelings in best interests decision-making.^92^ We are also under no illusions that the way in which mental capacity is considered—even by the courts—is always acceptable. And we are under no illusions that there are many circumstances in which failure to offer appropriate alternatives means that the person is left in a position where they have no choice but to accept or refuse a particular option. At that point, to ask whether the person has the capacity to do so may well be necessary, but is undoubtedly insufficient.^93^

We also recognise and indeed have emphasised, that mental capacity is a concept whose legitimacy is ultimately socially sustained. Put another way, if all of the processes that we have outlined in the paper for determining and responding to situations where a person appears to have difficulty making a decision were to be abolished,^94^ then it would be challenging to sustain a defence of the concept of mental capacity. Some might feel that it is so vital to challenge both the concept and the conditions for considering capacity that it is necessary, in effect, to pull down the house around them, in the hopes that a new one can be constructed. Others (and we belong here) may feel that the intellectual, emotional, and other resources of those concerned with securing the rights of those with impairments are better directed to testing, probing, and refining the processes necessary for a legitimate concept of mental capacity.

## Footnotes

1 The research team consists of an English psychiatrist, an Irish psychiatrist, a practising English barrister and academic, and an American bioethicist/psychiatrist. The research team regularly presented before the Service User Advisory Group appointed to stress-test the work of the overall project as well as to an annual interdisciplinary colloquium for the whole project.

2 Available at <https://capacityguide.org.uk/> accessed 2 December 2022.

3 Committee on the Rights of Persons with Disabilities, *General Comment No 1, Article 12: Equal recognition before the law* (UN, 2014) para 3 (General Comment 1).

4 We deliberately use the past tense here because the constitution of the Committee that published this General Comment is not the same as the constitution of the Committee at the time of writing, and, as suggested further below, there are signs that the more recent appointees to the Committee may not hold the same views as their predecessors.

5 ibid.

6 ibid, para 4, the Committee identifying that ‘[the] *present general comment reflects an interpretation of article 12 which is premised upon the general principles of the Convention*’. At para 12, the Committee identified that General Comment 1: *focuses primarily on the normative content of article 12 and the State obligations that emerge therefrom. The Committee will continue to carry out work in this area so as to provide further in-depth guidance on the rights and obligations deriving from article 12 in future concluding observations, general comments and other documents*.’

7 A detailed overview of the literature is provided in L Series and A Nilsson, ‘Article 12 CRPD: Equal Recognition before the law’ in I Banketas, M Ashley Stein and D Anastasiou (eds), *The UN Convention on the Rights of Persons with Disabilities: A Commentary* (OUP 2018).

8 The reference to ‘skill’ in General Comment 1 is ambiguous. It usually refers to something learned or acquired through training, which will always be a matter of degree. Further, in legislation such as the Mental Capacity Act 2005, ‘mental capacity’ is a term relating to legal status, as well as the person’s mental functioning. The more normal synonym for mental capacity would be ‘ability’: see, for instance, its use in the Australian Law Reform Commission report ‘Equality, Capacity and Disability in Commonwealth Laws’ (ALRC Report 124, August 2014) discussed below.

9 As an unincorporated international treaty, the CRPD does not form part of the law of the UK, and English courts cannot conclude that the UK is in breach of any obligations owed under the CRPD: see *R(SC, CB and 8 children) v Secretary of State for Work and Pensions & Ors* [2021] UKSC 26, [2021] 3 WLR 428 at paragraphs 77 and 84 and *A Local Authority v JB* [2021] UKSC 52 at para 120. The position adopted by the UK Supreme Court is that UN treaty body committees do not make binding decisions on the interpretation of ‘their’ treaty’ and (2) General Comments issued by such committees ‘have no defined status, and they are not analogous to the rulings of an international court’. *R(AB) v Secretary of State for Justice* [2021] UKSC 28, [2021] 4 WLR 494 at paras 64–65. The position of the Supreme Court in this latter regard is consistent with that adopted by the equivalent courts in other jurisdictions: see the judgment of the Second Senate of German Federal Constitutional Court in 2 BvR 309/15, judgment of 24 July 2018 at para 91: *according to Arts. 34 et seq. CRPD, the Committee has no mandate to issue binding interpretations of the text of the Convention. It is also not competent to further develop international conventions beyond the agreements and practices of the treaty parties (cf. Art. 31 of the Vienna Convention on the Law of Treaties of 23 May 1969, UNTS 1155, 331 <340>, BGBl II 1985 p. 926, affirming customary international law;* […]*). While its statements have significant weight, they are not binding on international tribunals or domestic courts* […].

10 [2021] EWHC 65 (Fam), [2021] 4 WLR 11.

11 See, for instance, *Re E (Medical treatment: Anorexia)* [2012] EWCOP 1639, [2012] Med LR 472 at paragraph 124 per Peter Jackson J (as he then was): ‘Article 8 ECHR guarantees the right to respect for a person's private life. This right belongs to *“everyone*” and the fact that a person lacks mental capacity does not deprive them of its protection’ (emphasis in the original).

12 In England and Wales, the precise mechanism by which this is achieved varies according to context, but in the context of acts in connection with care and treatment, it is achieved through the operation of section 5 Mental Capacity Act 2005, which ‘gives a general authority, to act in relation to the care or treatment of P, to those caring for him who reasonably believe both that P lacks [mental] capacity in relation to the matter and that it will be in P’s best interests for the act to be done’: *N v ACCG* [2017] UKSC 22 at paragraph 38. This achieves a *de facto* legal incapacitation of the person in respect of the decision because their purported refusal of the care would not be determinative; similarly, that they appeared to be assenting to it would not mean that they would be taken as having, legally, consented to it.

13 General Comment 1, para 14.

14 ibid, para 15.

15 Although whether it is the dominant discourse in such circles raises fascinating questions outside the scope of this article.

16 A particularly influential academic body being the National University of Ireland, Galway.

17 An influential pre-CRPD challenge to the concept of capacity was S Stefan ‘Silencing the different voice: Competence, feminist theory and law’ (1992–1993) 47 Miami Law Review 763. A fascinating—post-CRPD challenge—to the concept of capacity, sitting at a tangent to the Committee’s approach can be found in the work of Beverley Clough (see, in particular B Clough*, The Spaces of Mental Capacity Law: Moving Beyond Binaries* (Routledge 2021). Other challenges to capacity (perhaps more prevalent amongst those straddling academia and clinical/legal practice), which focused less not on the validity of the concept per se, and more on its formulation and application were also well established in the academic literature by the time that the CRPD was concluded. The potential for the application of the concept of mental capacity to be influenced by professional attitudes and/or ‘treatment bias’ was already familiar by the 1980s: see L Roth, A Meisel and C Lidz, ‘Tests of Competency to Consent to Treatment’ (1977) 134(3) American Journal of Psychiatry 279 and the discussion in the Law Commission’s first consultation paper: Law Commission, *Mentally incapacitated adults and decision-making: An Overview* (Law Com CP No 119, 1991), at para 2.38. The potential for the concept to be misapplied has been the subject of extensive examination, but for present purposes it is perhaps most useful to highlight the critical observations about the application of the mental capacity test in the post-legislative scrutiny report of the House of Lords Select Committee on the Mental Capacity Act 2005 (HL, 2014). We address our concerns about application in Section VI below.

18 [2021] UKSC 52.

19 See also A Ruck Keene and A Enefer, ‘What place has ‘capacity’ in the criminal law relating to sex post JB?’ (2022) 85 IJLP 101843.

20 Para 120; as noted at n 9 above, the Supreme Court also held that it would not, in any event, examine the question of whether the UK was in breach of the provisions of the CRPD, as an unincorporated international treaty.

21 See the decision of the *Bundesverfassunsgericht* (German Federal Constitutional Court) 1BVL 81/5 of 26 July 2016 explained in *Bundesverfassunsgericht*, ‘Limiting Coercive Medical Treatment to Persons under Custodianship Confined in Accommodations is Incompatible with the State’s Duty of Protection’ (Press release, 59/2016, 29 August 2016) 2(d) <https://www.bundesverfassungsgericht.de/SharedDocs/Pressemitteilungen/EN/2016/bvg16-059.html> accessed 2 December 2022.

22 See *AM-V v Finland* [2017] ECHR 273 at para 89.

23 See W Martin and S Gurbai, ‘Surveying the Geneva Impasse: Coercive Care and Human Rights’ (2019) 65 IJLP 117 and, in particular, n 6, noting how the Human Rights Committee's Concluding Observations have seemed to recognise the permissibility of guardianship arrangements whereas GC1 rejects them. See also the 2009 Human Rights Committee Concluding Observations on the Russian Federation, CCPR/C/RUS/CO/6, 18 December 2009, para 19(a)).

24 eg the legislature in Ireland, enacting The Assisted Decision-Making (Capacity) Act 2015.

25 There is, though, an extensive literature in relation to the CRPD Committee’s approach; this is usefully summarised, and framed, in M Scholten and J Gather, ‘Adverse Consequences of Article 12 of the UN Convention on the Rights of Persons with Disabilities for Persons with Mental Disabilities and an Alternative way Forward’ (2018) 44 JME 226–33. More broadly, we would also highlight the work of Mary Donnelly, in particular her article ‘Changing Values and Growing Expectations: The Evolution of Capacity Law’ (2017) 70(1) Current Legal Problems 305.

26 In this regard, we note that it is possible also to overdo the differences between reaching a conclusion that a person has or lacks capacity to make a decision and reaching a conclusion that a person has or does not have a specific physical disorder. By way of example, a widely used method for detecting tuberculosis is a chest X-ray. Repeated studies have shown significant variability in chest X-ray diagnosis of TB, with either moderate or just ‘fair’ inter-rater agreement (ie the rate of agreement between different professionals reviewing the same X-ray): see J Zellweger and others, ‘Intra-observer and Overall Agreement in the Radiological Assessment of Tuberculosis’ (2006) 10 The International Journal of Tuberculosis and Lung Disease 1123; further, there is also variability in TB diagnosis between radiologists in different countries: see S Sakurada and others, ‘Inter-rater Agreement in the Assessment of Abnormal Chest X-ray Findings for Tuberculosis between Two Asian Countries’ (2012) 12 BMC *Infectious Diseases* 1. Some of the approaches that are adopted to seek to reduce ‘noise’ in the physical health context (see D Kahneman, O Sibony and C Sunstein, *Noise: A Flaw in Human Judgment* (William Collins, London, 2021)), have echoes with the approaches that can be adopted to seek to reach more satisfactory capacity determinations, and remind us that, at least in some cases, identifying a problem in the physical health context is not merely a question of discovery, but of evaluation in the same way that it is in relation to mental capacity.

27 See eg I Álvarez Marrodán and others, ‘Limits on the Use of the MMSE for Assessment of Capacity to Consent for Treatment’ (2018) 32 The European Journal of Psychiatry 153.

28 The Law Commission’s initial consultation paper, grounding the project which led ultimately to the MCA 2005, addressed the issue that ‘judgments [of competency] reflect social considerations and societal biases as much as they reflect matters of law and medicine’ (citing from L Roth, A Meisel and C Lidz, ‘Tests of Competency to Consent to Treatment’ (1977) 134(3) American Journal of Psychiatry 279). As the Law Commission identified: ‘[t]here is no one “right” method of assessing capacity, but a broad consensus can be reached upon the test which should be applied and the main considerations which should be taken into account’. Law Commission, *Mentally incapacitated adults and decision-making: An Overview* (Law Com CP No 119, 1991), para 2.42.

29 [2021] UKSC 52.

30 At which point, no-one could seek to decide on his behalf to engage in sexual relations: see s 27(1)(b) Mental Capacity Act 2005.

31 By virtue of s 3(4) Mental Capacity Act 2005.

32 As noted above, Lord Stephens also crisply rejected a challenge that this approach was contrary to the CRPD.

33
*Re HD (Capacity to Engage in Sexual Relations)* [2021] EWCOP 15.

34 Intervening in the appeal before the Supreme Court in JB, for instance, were a charity, Respond, providing therapy and specialist support services to people with learning disabilities, autism or both who have experienced abuse, violence or trauma, and the Centre for Women’s Justice, a charity whose purpose is to bring strategic law challenges and ensure access to justice for victims of male violence. 

35 See further in this regard, N Banner, ‘Unreasonable reasons: normative judgements in the assessment of mental capacity’ (2012) 18 Journal of evaluation in clinical practice 1038.

36 The precise link between mental capacity and concepts of criminal responsibility is beyond the scope of this paper, but they both occupy the same conceptual space: see, for instance, the Law Commission’s 2013 discussion paper ‘Criminal Liability: Insanity and Automatism) (Law Com P No 2013, 1993), Chapter 4.

37 Indeed, equally philosophically inadequate—or least incoherent—might be thought to be the construct of human rights themselves. See R Cruft, S Liao and M Renzo, ‘The Philosophical Foundations of Human Rights: An Overview’ in R Cruft, S Liao and M Renzo (eds), *The Philosophical Foundations of Human Rights* (OUP 2015).

38 See in this regard—and for the concept of ‘incompletely theorised agreements’—the work of Cass Sunstein, especially C Sunstein, *Legal Reasoning and Political Conflict* (2nd edn, OUP 2018).

39 A Arstein-Kerslake, ‘Understanding Sex: The Right to Legal Capacity to Consent to Sex’ (2015) 10 Disability & Society 1459, 1469.

40 Tina Minkowitz, who is not a member of the Committee, but whose writings and work have been prominent in the ‘hard-line’ approach interpretation of Article 12 CRPD, has sought to do so. In an article available (it appears only in pre-print) on the SSRN, ‘CRPD Article 12 and the Alternative to Functional Capacity: Preliminary Thoughts Towards Transformation’, (25 December 2013) <https://papers.ssrn.com/sol3/papers.cfm?abstract_id=2371939> accessed 2 December 2022, she advances an argument that, in essence, where there are no power imbalances there is no need for a concept of functional mental capacity. It may have been this approach which influenced the approach taken the next year in General Comment 1.

41 This is a different point, we note, to the question of whether the Committee’s approach to supported decision-making provides sufficient safeguards against the potential for undue influence or exploitation in the support mechanisms proposed. This has provoked an extensive discussion in the literature (reviewed from a perspective sympathetic to the Committee) in P Gooding ‘Navigating the ‘flashing amber lights’ of the right to legal capacity in the United Nations Convention on the Rights of Persons with Disabilities: Responding to major concerns’ (2015) 15(1) Human Rights Law Review 45). Our point is as to the basis upon the Committee rejects the concept of mental capacity as a necessary component of legal capacity (at least in relation to adults).

42 In his contribution to M Ashley Stein and others (eds), *Mental Health, Legal Capacity and Human Rights* (CUP 2021) at 56. Neuman has previously written that ‘specialized treaty bodies may lack legal expertise [or] pay insufficient attention to the rights of others that may come into conflict with the rights within their specialized mandate’. See his contribution to D Moeckli, H Keller and C Heri (eds), *The Human Rights Covenants at 50: Their Past, Present, and Future* (OUP 2018), at page 42.

43 As Martin and Gurbai (n 22 above) note, the Human Rights Committee's Concluding Observations have seemed to recognise the permissibility of guardianship arrangements, based upon a link between mental disability and legal capacity, emphasising (in relation to Russia) that it should: [r]*eview its policy of depriving persons with mental disabilities of their legal capacity and establish the necessity and proportionality of any measure on an individual basis with effective procedural safeguards, ensuring in any event that all persons deprived of their legal capacity have prompt access to an effective judicial review of the decisions*. See (para 19(a) of the Concluding Observations, CCPR/C/RUS/CO/6, 24 November 2009; the more recent Concluding Observations, in 2015, do not address this issue).

44 See W Martin and S Gurbai, ‘Surveying the Geneva Impasse: Coercive Care and Human Rights’ (2019) 65 IJLP 117 and, in particular, n 8.

45 See, for a flavour of this, J Craigie and others (eds), ‘Legal Capacity, Mental Capacity and Supported Decision-making: Report from a Panel Event’ (2019) 62 IJLP 160, and in particular the observation by Craigie that: [f]*ollowing the discussion, a few audience members expressed concerns to the moderator and I that their perspectives as survivors of psychiatry had not been represented on the panel. The issue of representation seemed important to raise here because those concerns were strongly felt, but also because I believe that this is a central challenge for this area, which needs to be more broadly acknowledged and explored. The audience members' objection was not, as I understood it, that their views about mental and legal capacity were absent from the event, but rather that the service user voice on the panel did not represent their experience or interests*.

46 A Plumb, ‘UN Convention on the Rights of Persons with Disabilities: Out of the Frying Pan and into the Fire? Mental Health Service Users and Survivors Aligning with the Disability Movement’ in H Spandler, J Anderson and B Sapey (eds), *Madness, Distress and the Politics of Disablement* (Policy Press 2015).

47 We also note in this regard a telling footnote in the introduction to the work in which Neuman’s observation appears, a book edited by a team who could be described as generally supportive of the Committee’s approach. The focus of the essays is upon legal capacity in the context of mental health. However, in a footnote in the introduction (n 1, page 2) the editors identify that intellectual disabilities and degenerative conditions such as Alzheimer’s ‘are likely to be affected by changes in decision-making regimes and should, therefore, be considered in debates relating to legal capacity’. They continue that, while in practical terms such was not possible for the book to consider such situations: *it is conceivable that many of the findings and assumptions relating to mental health may apply to intellectual disabilities, dementia and other conditions which affect capacity. However, this is not a universal truth, and conclusions drawn here about the mental health care system should be interrogated further before being applied to social care models for the intellectually disabled or for those whose condition may not improve with time*.

48 Australian Law Reform Commission, ‘Equality, Capacity and Disability in Commonwealth Laws’ (ALRC Report 124, August 2014), para 3.45.

49 Reflecting the language of Article 12(4) CRPD, although it is striking how often the full phrase in Article 12(4), “**rights**, will and preferences” is not used. For instance, General Comment 1 notes (at paragraph 26): [i]*n its concluding observations on States parties’ initial reports, in relation to article 12, the Committee on the Rights of Persons with Disabilities has repeatedly stated that States parties must review the laws allowing for guardianship and trusteeship, and take action to develop laws and policies to replace regimes of substitute decision-making by supported decision-making, which respects the person’s* ***autonomy****, will and preferences.* (emphasis added).

50 Indeed, this is implicitly recognised by the group at the Centre for Disability Law and Policy (‘CDLP’) at the National University of Ireland, Galway, one of the main proponents of the ‘hard-line’ CRPD model. See their contribution to the Australian Law Reform Commission’s work noted at paragraph 3.86 of the ALRC’s 2014 Report (n 57 below) at para 3.86: *Many people with anorexia express a will to live, but a preference to not eat. In these cases, an outside decision-maker may be involved, but would still be restricted from making a decision that was contrary to the individual’s expressed will and preference. PEG feeding, for example, would only be allowed if the individual agreed to it. These situations will always be difficult—they are difficult under ‘best interests’ determinations and they will continue to be difficult under an approach that prioritises will and preference.* The CDLP submission as recorded in the ALRC’s Report does not propose a solution to this conundrum.

51 See, for a development of this theme, S Kim and A Ruck Keene, ‘A new kind of paternalism in surrogate decision-making? The case of Barnsley Hospitals NHS Foundation Trust v MSP’ (2021) 47(12) Journal of Medical Ethics e81.

52 CRPD/C/AUS/CO/2-3, 15 October 2019.

53 ALRC Report 124 August 2014.

54 What that latter phrase means is fleshed out at para 3.83, which provides that: *3.83 The human rights approach is also reflected in the paragraph 2(d) of the Will, Preferences and Rights Guidelines* [one of a set of guidelines for framing law reform proposed by the ALRC]*, which provides that a representative may override the will and preferences of a person only where necessary to prevent harm. This is consistent with the CRPD in that, for example, art 17 of the CRPD may require the representative to make a decision that protects the person’s ‘physical and mental integrity’, notwithstanding the decision conflicts with the person’s expressed will and preferences. A qualification of this kind tests the limits of autonomy, particularly where the limitation concerns harm to oneself.* […] *3.84 A provision that a person’s will and preferences may be overridden based on the outcome of a decision—in this case, harm—runs contrary to a focus on ability that is not outcomes-based. [..] However, it is not necessarily inconsistent with a principle of autonomy, as autonomy is not an absolute concept*.

55 Professor Theresia Degener: editor’s foreword to the special issue of (2017) 13 International Journal of Law in Context 1.

56 Who, subsequent to the completion of the report upon Australia, was appointed Chair of the CRPD Committee.

57 Which does not govern all situations where a person’s legal capacity to make a decision or take an action may be in question: common law tests apply to such matters as making a will (*Banks v Goodfellow* (1870) LR 5 QB 549), making a gift (*Re Beaney* (Deceased) [1978] 2 All ER 595) or entering into a contract (*Boughton v Knight* (1873) LR 3 PD 64). Other statutory tests apply, for instance, to the question of whether a person can consent to sexual relations for purposes of the potential commission of the offence of rape under the Sexual Offences Act 2003: see *R v Cooper (Gary Anthony)* [2009] 1 WLR 1786.

58 See also the decision of the Supreme Court in *A Local Authority v JB* [2021] UKSC 52, in which Lord Stephens emphasised the need for the identification of a ‘clear causative nexus between P’s inability to make a decision for himself in relation to the matter and an impairment of, or a disturbance in the functioning of, P’s mind or brain*’*.

59 Law Commission, *Mentally incapacitated adults and decision-making: Medical treatment and research* (Law Com CP No 129, 1993), paras 3.10–3.14.

60 Law Commission, *Mental Incapacity* (Law Com No 231, 1995), para 3.8.

61 The arguments are traversed in W Martin and others, *The Essex Autonomy Project Three Jurisdictions Report: Towards Compliance with CRPD Art 12 in Capacity/Incapacity Legislation across the UK* (University of Essex 2016).

62 The Assisted Decision-Making (Capacity) Act 2015, which provides (at section 3) a model of mental capacity which looks very similar to that contained in the Mental Capacity Act 2005, ss 2 and 3, but without any requirement that the functional inability to make the decision in question be caused by an impairment or disturbance in the person’s mind or brain.

63 Decision Support Service: Code of Practice on Supporting Decision-making and Assessing Capacity (15 November 2021), available at <https://decisionsupportservice.ie/sites/default/files/2021-11/DSS-COP_SDM-and-Capacity-final.pdf> accessed 2 December 2022.

64 Our research suggests that even where no requirement appears in statute, it is likely to be read in by those operating it. This certainly appears to have been the case in New Zealand, where our interviews with practising lawyers highlighted to us the fact that, whilst the governing legislation (for these purposes), the Protection of Personal and Property Rights Act 1988, includes no such ‘diagnostic’ element, in practice proceedings cannot be brought absent evidence of such an element in play. The implementation of the Irish legislation may provide the opportunity for further fruitful research in this respect.

65 See the impact log report for the SUAG meeting of 21 June 2018, available at <https://mhj.org.uk/wp-content/uploads/2021/09/MHJ-SUAG-collated-impact-log-data-forms-Version-2-17Aug21.docx> accessed 2 December 2022.

66 The term routinely used in relation to the Mental Capacity Act 2005, even if inaccurately, there being no requirement of formal ‘diagnosis’.

67 See, in particular, the decision of Munby J in *A Local Authority v (1) MA (2) NA and (3) SA* [2005] EWHC 2942, [2006] 1 FLR 867, that of the Court of Appeal in *DL v A Local Authority and Others* [2012] EWCA Civ 253, [2012] COPLR 504 and the lecture of Sir James Munby to the Court of Protection Bar Association on 10 December 2020 <https://www.cpba.org.uk/wp-content/uploads/2020/12/2020COPBA.pdf> accessed 2 December 2022. See also L Pritchard-Jones, ‘The Good, the Bad, and the “Vulnerable Older Adult”’ (2016) 38(1) Journal of Social Welfare and Family Law 51 and J Herring, *Vulnerable Adults and the Law* (OUP 2016).

68 In this, and acknowledging that there is a deep hinterland of literature about the concepts, we draw upon the work of M Burch and K Furman, ‘Objectivity in Science and Law: A Shared Rescue Strategy’ (2019) 64 IJLP 60.

69 See Baker J (as he then was) in *Re TZ (No 2)* [2014] EWHC 973 (COP), [2014] COPLR 159 at paragraph 28: ‘In a case involving a vulnerable adult, there is a risk that all professionals involved with treating and helping that person—including, of course, a judge in the Court of Protection—may feel drawn towards an outcome that is more protective of the adult and thus, in certain circumstances, fail to carry out an assessment of capacity that is detached and objective.’

70 A Ruck Keene, A ‘Is mental capacity in the eye of the beholder?’ (2017) 11(2) Advances in Mental Health and Intellectual Disabilities 30-39.

71 ie the Special Rules for Terminal Illness which fast-tracked benefit applications for those with a terminal diagnosis of six months, which the Government announced in July 2021 were to be replaced with a new 12-month, end of life definition. DWP ‘Fast-tracked access to benefits for people with terminal illness expanded’ <https://www.gov.uk/government/news/fast-tracked-access-to-benefits-for-people-with-terminal-illness-expanded> accessed 2 December 2022. Ironically, at almost exactly the same time, a Bill was advanced before Parliament relying upon the same prognostic ability as the ‘entry point’ to obtaining medical assistance with dying: see the Assisted Dying Bill introduced by Baroness Meacher which links (in Clause 2) the concept of ‘terminal illness’ to the diagnosis of an inevitably progressive condition which cannot be reversed by treatment, in consequence of which the person is reasonably expected to die within six months.

72 [2019] EWCA Civ 872, [2020] 4 WLR 38. In overturning the decision (on a different point), the Supreme Court ([2021] UKSC 38) identified that the obligation of immigration officers ‘is to apply the statute in accordance with its terms according to their best judgment, formed in the light of the available evidence’ (para 67).

73 It is perhaps also worth noting here that even where a date of birth is given, this does not, in fact, necessarily determine a person’s age for all purposes: the age for a person adopting the traditional Korean method of calculating age is not the same as for a person using a Western model. Even in the context of something which is a ‘naturally objective, scientific and naturally occurring phenomenon’, therefore, it is a phenomenon which is in part based upon a social construction.

74 para 52.

75 Difficulties which the Westminster Government at the time of writing is proposing to address on the basis that—contrary to the position in *BF—*it is possible to use ‘scientific methods’: see Home Office, *New Plan for Immigration: policy statement* <https://www.gov.uk/government/consultations/new-plan-for-immigration/new-plan-for-immigration-policy-statement-accessible> accessed 2 December 2022. Committee on the Rights of Persons with Disabilities, *General Comment No 1, Article 12: Equal recognition before the law* (UN, 2014) para 3 (General Comment 1).

76 After the decision in *R (B) v London Borough of Merton* [2003] EWHC 1689 (Admin), [2003] 4 All ER 280. In clinical terms, they would be described as structured assessments, undertaken over a period of time, and triangulating self-reporting against any available corroborative information.

77 fn 5 to para 56 of *BF*. At fn 7, Underhill LJ noted that: *even a Merton-compliant assessment does not represent objective fact* […] *the Appellant in this case had had three apparently Merton-compliant assessments and that it was only in the third that he was found to be a child*.

78 See, for instance, J Kassirer, J Wong and R Kopelman, *Learning Clinical Reasoning* (2^nd^ revised edition, Lippincott Williams and Wilkins 2009).

79 [2018] VSC 564.

80 Whilst on its face relating to medical treatment, this paragraph applies equally to other areas—for instance the right not to be required to live at a place one does not wish.

81 In the context of consideration of best interests—i.e. the test to apply where the person does not have capacity to make the decision—District Judge Eldergill has pithily observed that: [t]*he law requires objective analysis of a subject not an object. The incapacitated person is the subject. Therefore, it is their welfare in the context of their wishes, feelings, beliefs and values that is important. This is the principle of beneficence which asserts an obligation to help others further their important and legitimate interests, not one's own. In this important sense, the judge no less than the public authorities is* [the person’s] servant, *not his master*’ (*Re AM, residence and care of*) [2019] EWCOP 59, in a judgment without paragraph numbers).

82 There are potentially other considerations in play when it comes to the interests of others. As identified by the Supreme Court in *A Local Authority v JB* [2021] UKSC 52, endorsing the observation of the Court of Appeal at para 98 of its judgment: ‘[a]lthough the Court of Protection’s principal responsibility is towards P, it is part of the wider system of justice which exists to protect society as a whole’.

83 This applies just as much to the CRPD as it does to the ECHR, the CRPD including a right to life (art 10) which derives from the right to life contained in art 6 of the International Covenant on Civil and Political Rights, itself containing a positive obligation to secure life: see Human Rights Committee, *General Comment No 36 on art 6 of the International Covenant on Civil and Political Rights, on the right to life* (UN, 2018), especially paras 7, 9, 21 and 24. See also K Wilson, ‘The Call for the Abolition of Mental Health Law: The Challenges of Suicide, Accidental Death and the Equal Enjoyment of the Right to Life’ (2018) 18 HLRLR 651.

84 See, for a neat encapsulation of the position in respect of life-saving medical treatment, *Kings College Hospital NHS Foundation Trust v C* [2015] EWCOP 80, [2016] COPLR 50.

85 In the form of research-informed guidelines available at <https://capacityguide.org.uk/> accessed 2 December 2022.

86 See the references at n 1.

87 Questions which one retired judge has posed in elegantly clear and penetrating fashion: M Hedley, *The Modern Judge: Power, Responsibility and Society's Expectations* (Jordan Publishing 2016).

88 Exceptions include: (i) where a person is discharging a statutory function under the Mental Capacity Act 2005 (for instance determining whether the person satisfies the relevant capacity requirement for purposes of the deprivation of liberty authorisation regime contained at the time of writing in Schedule A1 to the Act); (ii) where the question is not one of care or treatment; or (iii) where the question of capacity is not governed by the Mental Capacity Act 2005 but by the common law (for instance, whether the person had capacity to make a will in the context of a post-death challenge to its validity).

89 A search of ‘paradigm shift’ AND ‘Convention on the Rights of Persons with Disabilities’ on Google Scholar produces almost 5,000 results <accessed 4 December 2022>.

90 See T Kuhn, *The Structure of Scientific Revolutions* (Columbia University Press 1962).

91 Although pre-dating it: s 1(3) Mental Capacity Act 2005 provides that ‘[a] person is not to be treated as unable to make a decision unless all practicable steps to help him to do so have been taken without success’.

92 It is, perhaps, appropriate to note that this course of action is not, itself, without peril if carried out without sufficient attention to its implications: see S Kim and A Ruck Keene, ‘A new kind of paternalism in surrogate decision-making? The case of Barnsley Hospitals NHS Foundation Trust v MSP’ (2021) 47(12) Journal of Medical Ethics e81.

93 For a stimulating challenge here, see B Clough*, The Spaces of Mental Capacity Law: Moving Beyond Binaries* (Routledge 2021).

94 And/or all of the functions that we have identified the concept of capacity serves were no longer to be accepted as socially legitimate.

